# Polygenic risk provides biological validity for the ICHD-3 criteria among Finnish migraine families

**DOI:** 10.1177/03331024211045651

**Published:** 2021-10-14

**Authors:** Paavo Häppölä, Padhraig Gormley, Marjo E Nuottamo, Ville Artto, Marja-Liisa Sumelahti, Markku Nissilä, Petra Keski-Säntti, Matti Ilmavirta, Mari A Kaunisto, Eija I Hämäläinen, Samuli Ripatti, Matti Pirinen, Maija Wessman, Aarno Palotie, Mikko Kallela, International Headache Genetics Consortium (IHGC)

**Affiliations:** 1 Institute for Molecular Medicine Finland FIMM, HiLIFE, University of Helsinki, Helsinki, Finland; 2 GlaxoSmithKline, Cambridge, MA, USA*; 3 Department of Neurology, Massachusetts General Hospital, Boston, MA, USA; 4 Psychiatric and Neurodevelopmental Genetics Unit, Massachusetts General Hospital, and Harvard Medical School, Boston, MA, USA; 5 Program in Medical and Population Genetics, The Broad Institute of MIT and Harvard, Cambridge, MA, USA; 6 The Stanley Center for Psychiatric Research, The Broad Institute of MIT and Harvard, Cambridge, MA, USA; 7 Folkhälsan Research Center, Helsinki, Finland; 8 Department of Neurology, Helsinki University Hospital and University of Helsinki, Finland; 9 Faculty of Medicine and Health, University of Tampere, Tampere, Finland; 10 Terveystalo Clinical Research, Turku, Finland; 11 Terveystalo, Helsinki, Finland; 12 Department of Neurology, Central Hospital Central Finland, Jyväskylä, Finland; 13 Department of Public Health, Clinicum, University of Helsinki, Helsinki, Finland; 14 Department of Mathematics and Statistics, University of Helsinki, Helsinki, Finland; 15 Analytic and Translational Genetics Unit, Department of Medicine, and Department of Psychiatry, Massachusetts General Hospital, Boston, MA, USA

**Keywords:** Migraine, headache, diagnosis criteria, polygenic risk score

## Abstract

**Background:**

Migraine is diagnosed using the extensively field-tested International Classification of Headache Disorders (ICHD-3) consensus criteria derived by the International Headache Society. To evaluate the criteria in respect to a measurable biomarker, we studied the relationship between the main ICHD-3 criteria and the polygenic risk score, a measure of common variant burden in migraine.

**Methods:**

We used linear mixed models to study the correlation of ICHD-3 diagnostic criteria, underlying symptoms, and main diagnoses with the polygenic risk score of migraine in a cohort of 8602 individuals from the Finnish Migraine Genome Project.

**Results:**

Main diagnostic categories and all underlying diagnostic criteria formed a consistent continuum along the increasing polygenic burden. Polygenic risk was associated with the heterogeneous clinical picture starting from the non-migraine headache (mean 0.07; 95% CI 0.02–0.12; *p* = 0.008 compared to the non-headache group), to probable migraine (mean 0.13; 95% CI 0.08–0.18; *p* < 0.001), migraine headache (mean 0.17; 95% CI 0.14–0.21; *p* < 0.001) and migraine with typical visual aura (mean 0.29; 95% CI 0.26–0.33; *p* < 0.001), all the way to the hemiplegic aura (mean 0.37; 95% CI 0.31–0.43; *p* < 0.001). All individual ICHD-3 symptoms and the total number of reported symptoms, a surrogate of migraine complexity, demonstrated a clear inclination with an increasing polygenic risk.

**Conclusions:**

The complex migraine phenotype progressively follows the polygenic burden from individuals with no headache to non-migrainous headache and up to patients with attacks manifesting all the features of the ICHD-3 headache and aura. Results provide further biological support for the ICHD-3 diagnostic criteria.

## Introduction

The current understanding is that migraine is a neurovascular disorder characterised by neuronal aura symptoms and vascular headache (1). Since 1988, migraine has been defined by the criteria set by the Headache Classification Committee of the International Headache Society (IHS). ICHD criteria (2) have been an improvement for migraine research (3,4) and have been shown to perform well both in the scientific research and in the clinic (5,6).

Since criteria are based on expert opinion, although extensively field-tested, the correlation with measurable biological markers would naturally be of interest. To evaluate the criteria in this regard, we set out to study the correlation between the current ICHD-3 criteria and the polygenic risk score (PRS) of migraine. PRS is a genetic biomarker constructed by combining effects of numerous genetic markers identified to associate with the disease in large genome-wide association studies (GWAS) (7–9). A PRS of 38,872 common variants is used here as the index of known molecular genetic predisposition to migraine with or without aura. We investigate its relationship to the main ICHD-3 diagnostic criteria in a study population of over 600 migraine families and 8602 individuals from the Finnish Migraine Genome Project.

## Methods

### Participants and migraine diagnosis

The Finnish Migraine Genome Project (FMGP) is a family study where families with at least four members affected by migraine are collected and analysed. The participants are recruited from patients attending headache clinics in Helsinki, Turku, Jyväskylä, Tampere, and Kemi in Finland. After being clinically diagnosed by a neurologist as suffering from migraine and consenting to participate, the patient (i.e. the index case) contacts all the other members of the family believed to suffer from migraine and asks whether they would be willing to participate in the study. If at least three possible migraineurs are willing to take part, the previously validated migraine specific questionnaire, the Finnish Migraine specific questionnaire for family studies (FMSQFS) (10) is mailed to each of them, and also to their parents and siblings regardless of their migraine status. Each questionnaire is then analysed in detail by the study neurologist.

The study questionnaire has more than 100 questions considering headache, aura, prodromal symptoms, other headache-associated symptoms, and comorbidities. Patients can describe their headache symptoms in multiple-choice questions and words, and aura symptoms in words, multiple-choice questions, and drawings. All diagnoses are made by a study neurologist based on the full questionnaire by following the ICHD-3 criteria. In case of uncertainty about the diagnosis, subjects are contacted by phone or at the office for detailed interview. The questionnaire has been previously validated for migraine without aura (MwoA) and migraine with aura (MwA), for both sensitivity and specificity (10). For this study, we included three additional classifications that were reasonable to derive with precision from our data: “Probable migraine” for participants reporting all but one of the necessary migraine diagnostic criteria, “Headache” for those having headache but missing more than one of the criteria, and “No headache” for individuals who did not report headache at all. Unanswered questions were interpreted as no-responses. Main diagnostic criteria (A–D) investigated in this study are listed in Supplemental Table 1.

**Figure 1.**
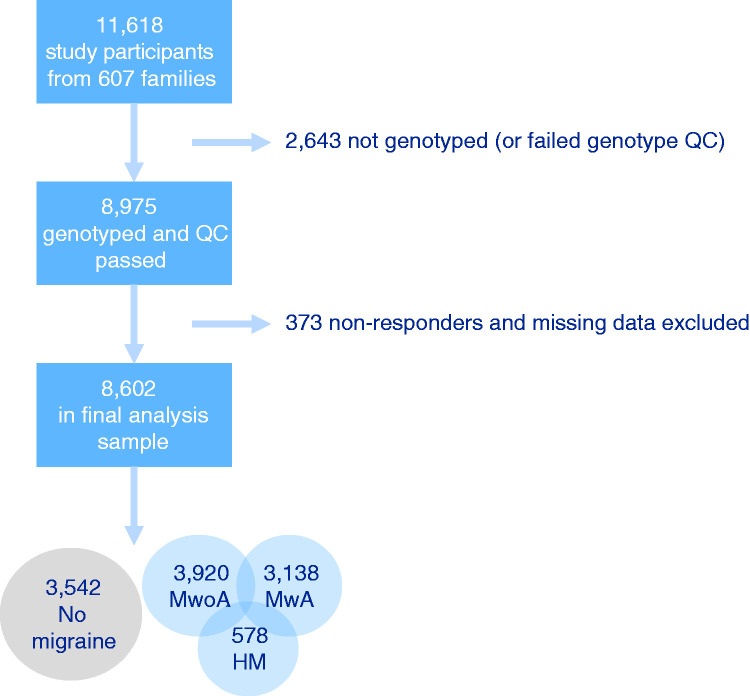
Flowchart of study participants. Diagnoses are not mutually exclusive. MwoA: migraine without aura; MwA: migraine with aura; HM: hemiplegic migraine.

The database freeze was in June 2020, having 607 migraine families, 7292 migraine cases, and 11,618 participants in total. For this study, we included a subset of 8602 individuals who had been genotyped and had reliable response data to study all symptoms of interest (Figure 1 and Table 1).

**Table 1.** Study participants. Categories are not mutually exclusive.

Participants (%)	N(women/men)	Mean age at response ± sd(women/men)
All participating family members	8602 (5611/2991)	46.1 ± 18.3(46.1/46.1)
HM cases (6.7%)	578 (487/91)	41.9 ± 14.5(42.6/38.3)
MwA cases (36.5%)	3138 (2405/733)	47.8 ± 16.5(47.7/47.8)
MwoA cases (45.6%)	3920 (3190/730)	44.4 ± 16.5 (44.8/42.8)
Probable migraine cases (19.0%)	1634 (1060/574)	45.1 ± 18.2(46.1/43.1)
Headache cases (13.8%)	1191 (667/524)	46.7 ± 20.2(48.2/44.8)
No headache (21.6%)	1857 (694/1163)	50.0 ± 19.2(49.6/50.2)

### Genotyping, imputation, and polygenic risk scores

We used polygenic risk scores derived in Gormley et al. (8). Samples were genotyped using Illumina CoreExome and Illumina PsychArray that share a common Infinium HumanCore backbone of 480,000 variants. Cases and controls were distributed evenly across batches. Standard GWAS quality control protocols (11) were conducted twice (batch-wise before merging and over the whole study after merging) as elaborated in the Supplementary Appendix.

After QC and merging, genotypes were phased with SHAPEIT2 (12) using the duoHMM algorithm to leverage the available pedigree structure in the family study, and then imputed with IMPUTE2 (13) using a Finnish reference panel consisting of 1940 whole genome sequences (WGS) combined with 1540 whole exome sequences (WES).

Polygenic risk score was constructed using SNP effect size estimates of common variants in the latest published GWAS of migraine in 375,000 individuals (14) by first excluding all Finnish studies. Score was then calculated with LD-clumping and P-thresholding approach as a sum of alleles weighted by their effect size estimates in the GWAS, resulting in 38,872 variants in the final score (technical details in the Supplementary appendix). The PRS distribution of the family study is originally scaled and centered to the mean and standard deviation of the National FINRISK study (15), giving it an interpretable scale with respect to the general population. Zero reflects the mean of the migraine PRS in the Finnish population, and one reflects the population standard deviation.

### Statistical analysis

To account for the high degree of relatedness in the family data, PRS distributions between groups were compared using linear mixed models where the empirical genetic relatedness matrix (GRM) was included as a random effect. GRM was calculated from an LD-pruned independent set of common SNPs. All statistical analyses were implemented with R-3.5.3 (16) and GMMAT package (17).

We first inspected variables of interest with a univariate approach and then combined them into a multivariable model to address their mutual correlation. Univariate results are reported as mean responses and multivariable results as effect estimates. *p*-values were derived using a Wald test. Multiple testing in univariate analyses was taken into account with Holm-Bonferroni correction.

## Results

Table 2 shows the proportion of the patients fulfilling each ICHD-3 main criteria (A–D) or having individual diagnostic symptoms in each of the diagnostic categories.

**Table 2.** ICHD-3 criteria and individual symptoms by diagnostic category and sex (women/men).

Headache variable	Headache %n = 1191(W/M)	Probable migraine %n = 1634(W/M)	MwoA %n = 3920(W/M)	MwA %n = 3138(W/M)	HM %n = 578(W/M)
*Main diagnostic criteria*					

Criterion A (number of attacks)	74.0(70/79)	77.0 (70/89)	100 (100/100)	87.7 (84.3/88.7)	96.5 (93.4/97.1)
Criterion B(headache duration)	14.4 (17.4/10.7)	50.6 (54.4/43.6)	100 (100/100)	75.7 (58.4/81.0)	88.4 (80.2/89.9)
Criterion C(headache characteristics)	33.8 (36.1/30.9)	85.6 (87.1/82.8)	100 (100/100)	86.6 (74.8/90.2)	95.6 (91.2/96.5)
Criterion D(nausea and sensory sensitivity)	41.9(43.9/39.3)	87.1 (88.1/85.2)	100 (100/100)	89.2 (80.1/91.9)	97.8 (95.6/97.5)
Criterion D1(nausea or vomiting)	34.2 (36.0/31.9)	75.7 (77.6/72.1)	92.7 (93.6/88.9)	82.4 (71.4/85.7)	91.7 (83.5/93.2)
Criterion D2(sensory sensitivity)	22.3 (25.0/31.9)	58.4 (61.2/53.3)	84.8 (87.5/73.0)	77.7 (58.1/79.8)	90.1 (85.7/91.0)

*Individual symptoms*					

Unilaterality	28.4 (31.5/24.4)	53.2 (57.2/45.8)	76.4 (79.3/63.4)	68.2 (50.6/73.5)	84.4 (71.4/86.9)
Pulsating	25.9 (27.7/23.5)	56.1 (58.9/50.9)	68.8 (69.7/64.9)	59.4 (47.5/63.0)	67.7 (63.7/68.4)
Moderate or severe	56.1 (58.8/52.7)	88 (89.4/85.4)	96.7 (97.1/94.9)	88.4 (78.9/91.4)	95.3 (93.4/95.7)
Physical activityaggravates	23.8 (24.1/23.5)	60 (61.2/57.8)	82.8 (83.7/78.6)	69.7 (57.3/73.5)	81.5 (72.5/83.2)
Nausea	33.8 (35.5/31.5)	75.3 (77.4/71.6)	92.4 (93.2/88.6)	82.1 (71.4/85.4)	91.5 (83.5/93.0)
Vomiting	16 (15.7/16.2)	40.9 (41.6/39.5)	63.6 (65.4/56.0)	56.2 (47.2/58.9)	69.6 (63.7/70.6)
Photophobia	49.2 (52.0/45.6)	80 (80.0/80.0)	95 (96.0/90.8)	89.2 (80.2/91.9)	97.1 (95.6/97.3)
Phonophobia	31.2 (34.6/26.9)	64.4 (68.3/57.3)	86.4 (89.1/74.5)	76.3 (59.9/81.3)	91.2 (86.8/92.0)

### Migraine diagnosis and PRS

**Figure 2.**
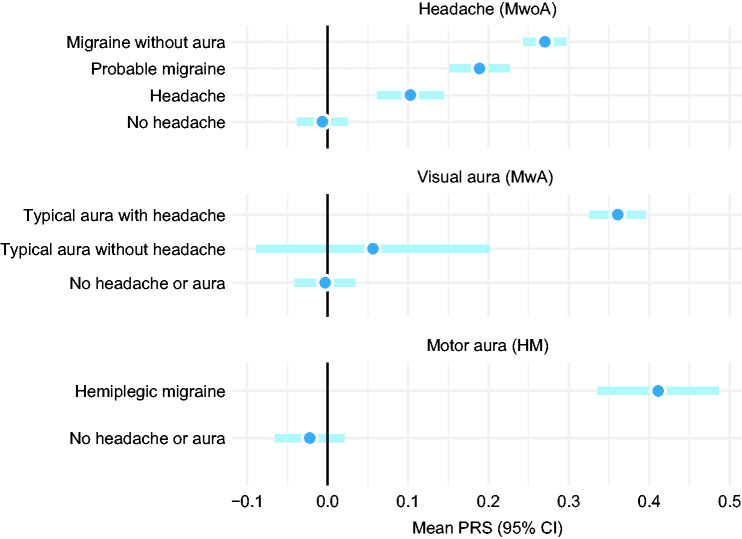
ICHD-3 diagnostic categories and mean polygenic risk score with 95% confidence intervals. PRS is scaled with respect to the mean and standard deviation of the general population.

**Figure 3.**
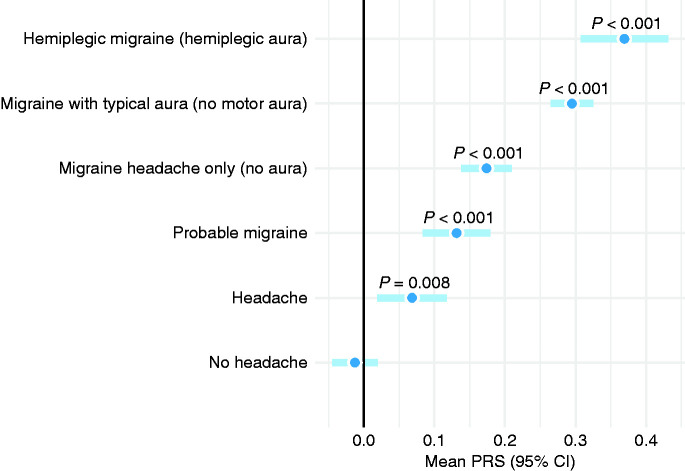
Migraine complexity continuum and mean PRS. Categories illustrated are mutually exclusive and all *p*-values contrasted to the “No headache” category.

Polygenic risk score correlated consistently along the migraine symptom complexity spectrum from “No headache” all the way up to “Hemiplegic migraine” (Figure 2 and Figure 3). Sub-diagnostic “Headache” and “Probable migraine” categories ranked below MwoA and MwA in terms of their mean PRS. Participants in these two categories report some migraine symptoms but not all necessary for an ICHD-3 migraine diagnosis. Interestingly, headache-free family members seem to have, on average, polygenic risk comparable to the general population mean.

### Main ICHD-3 criteria for headache

**Figure 4.**
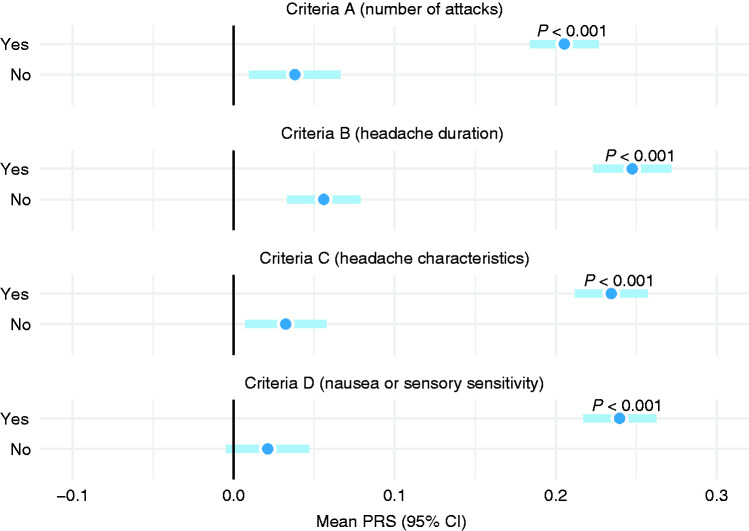
Main ICHD-3 criteria and mean PRS among all study participants.

Across the main ICHD-3 diagnostic criteria, criterion D (nausea and vomiting) demonstrated the strongest association with the PRS in our study (Figure 4) and criterion A (number of attacks) the least, when each criterion was assessed individually. Overall, the differences were very subtle, and all four criteria seem to correlate with the polygenic burden in a consistent manner. We did not find any significant differences among sexes in regard to polygenic risk across the four main criteria (A–D).

**Figure 5.**
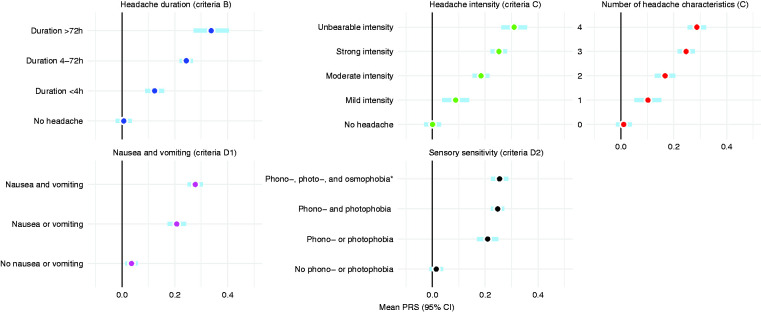
Mean PRS by strength of individual symptoms in the ICHD-3 criteria. *Osmophobia not included in the ICHD-3.

When dissecting criterion C (headache characteristics) into individual symptoms, we saw a clear connection in the number of reported criterion C symptoms and increased PRS (Figure 5). The trend was consistent even when we looked at the reported grade of headache intensity and duration. The more severe, long-lasting, and typical the reported headache, the higher the PRS was on average.

Reporting both nausea and vomiting together associated with a higher polygenic risk than either alone. Interestingly, the same was not apparent for photophobia and phonophobia, where one symptom clearly correlated with increased PRS but reporting both did not show a considerable additional increment. When we experimentally included osmophobia during the attack (18) (not part of the ICHD-3) as a third symptom among the D2 criteria (sensory sensitivity), we did not see a substantial increase in the mean PRS on top of photo- and phonophobia.

### Migraine complexity measured as the number of reported symptoms

**Figure 6.**
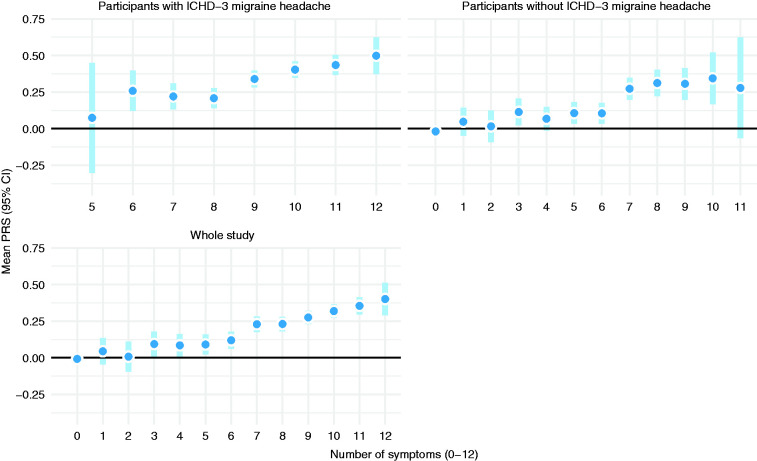
Mean PRS and migraine symptom complexity, measured by the number of reported diagnostic symptoms. A sum of 10 headache symptoms, visual aura, and hemiplegic aura.

Looking at individual symptoms underlying the main ICHD-3 criteria, we could see a consistent increasing trend in the underlying PRS and number of symptoms reported among all study participants (Figure 6). Interestingly, we saw a jump in the mean PRS when moving from six to seven reported symptoms, roughly at the same point where MwoA diagnosis could be considered if we just mechanistically examined criteria A–D. The same trend of increasing migraine symptom complexity (measured as a number of reported symptoms) along with PRS was seen among diagnosed participants. Interestingly, the signal is visible even among those who reported some symptoms but did not meet all the requirements for a migraine headache diagnosis.

### Aura characteristics among individuals with migraine headache

**Figure 7.**
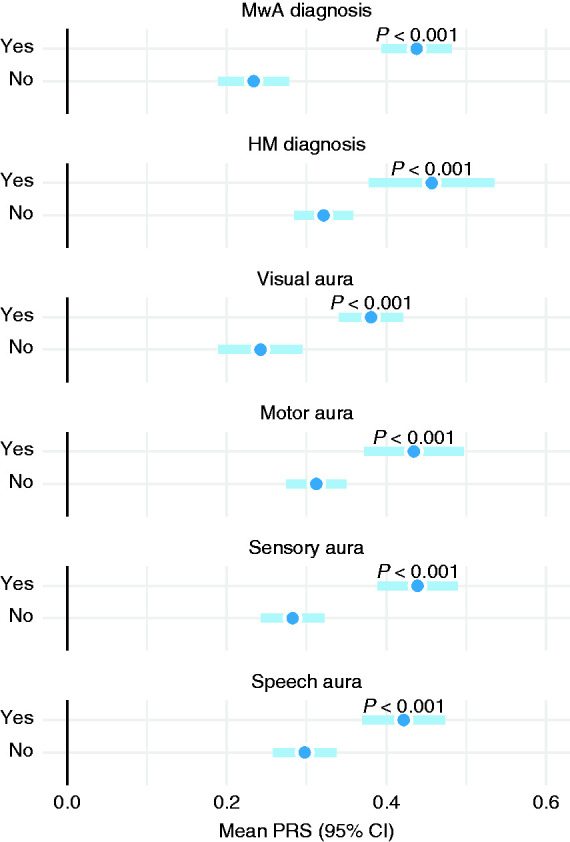
Mean PRS and aura characteristics among study participants with ICHD-3 migraine headache diagnosis.

Focusing on individuals whose headache fulfilled the ICHD-3 criteria, those reporting visual aura had a higher PRS on average compared to those only reporting headache (Figure 7). When different auras were considered individually, PRS seemed to contribute rather similarly to visual, sensory, speech, and motor aura.

### Ranking main criteria in respect to PRS

**Figure 8.**
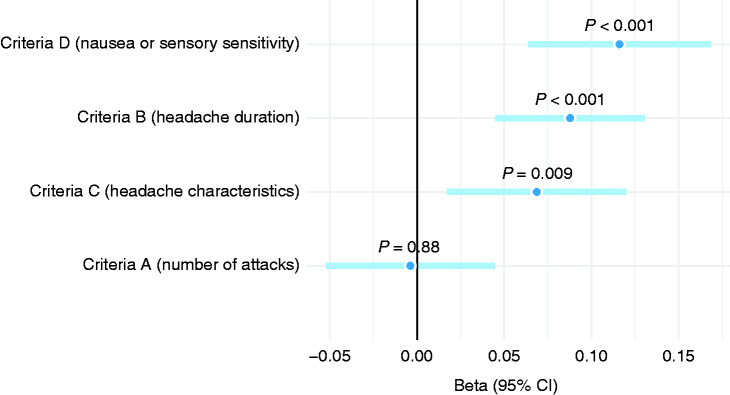
Main ICHD-3 diagnostic criteria in relation to PRS. Effect estimates (beta) for dichotomous criteria from a multivariable linear mixed model.

To address the mutual correlation between ICHD-3 MwoA diagnostic criteria, we evaluated all simultaneously in a multivariable model. This allowed us to inspect how they associated with the polygenic risk while adjusting for other criteria (Figure 8) and rank them by the strength of the association. Criterion A (number of attacks) contributed very little when all other criteria (B–D) were controlled for, whereas criterion D, including D1 (nausea and vomiting) and D2 (photo- and phonophobia) was the most important, as could be expected. Criterion B (headache duration) and criterion C (headache quality) also demonstrated association with polygenic risk after considering all other criteria.

**Figure 9.**
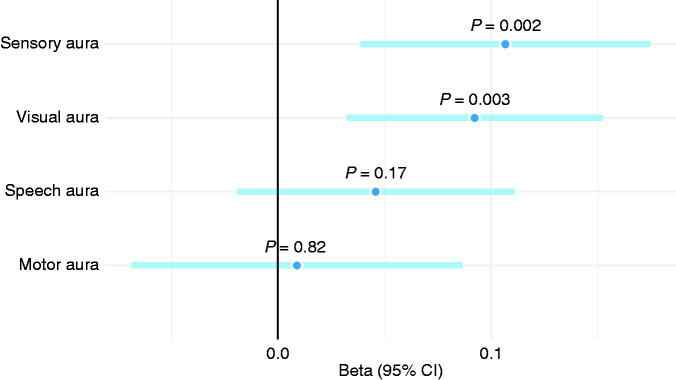
Aura characteristics in relation to PRS among participants with ICHD-3 migraine headache. Effect estimates (beta) from a multivariable linear mixed model.

To inspect how PRS correlates beyond the headache dimension of migraine, another multivariable model was built for ICHD-3 aura by considering only participants whose headache fulfilled the ICHD-3 diagnosis. Sensory and visual auras demonstrated the strongest association with the PRS (Figure 9) and the speech aura seemed to have a small effect on PRS, although not statistically significant. Motor aura was no longer associated with the PRS when the model was adjusted for the other three auras.

## Discussion

Our study of 8602 members from Finnish migraine families demonstrates that all the main ICHD-3 criteria and underlying individual diagnostic symptoms align well with one of the rare biomarkers we currently have for migraine (19), the polygenic risk score. Although polygenic risk explains only a small fraction of liability of complex diseases such as migraine (9,20) and cannot be considered as the ultimate biological reference measure for accuracy of any clinical criteria, we can reasonably say that the ICHD-3 consensus criteria clearly have a biological underpinning.

In our study, diagnostic categories formed a logical and consistent continuum along the increasing polygenic burden. The more typical and severe the migraine, the stronger correlation we saw with the polygenic risk score. Strikingly, the correlation generalised even outside the case-control definition of the original GWAS, demonstrating an analogous trend from sub-diagnosed headache all the way up to migraine with aura and hemiplegic migraine. Similarly, the number of reported symptoms, a surrogate of migraine complexity, demonstrated a clear inclination with increasing PRS, which occurred even among individuals who did not fulfill a migraine diagnosis but reported some headache symptoms.

We find it plausible that most participants in the sub-diagnostic “Headache” category may have tension-type headaches. It is noteworthy how well these non-migraine headache categories fit in the polygenic spectrum. This provokes interesting future research questions: Investigating the polygenic nature of migraine among tension-type headache might have both research and clinical value, because the genetic backbone of tension-type headache is still largely unknown and the link with migraine might be stronger than expected (21).

Notably, participants having a combination of ICHD-3 aura and the most symptoms of migraine headache had the highest PRS on average. This is thought-provoking, given that clinically headache is often very typical among patients with MwA and more so in the hemiplegic migraine (HM), although these diagnoses do not require a certain type of headache after the aura per se.

Our results show that migraine PRS aligns with the heterogeneous clinical picture of migraine starting from the sub-diagnostic headache, nausea, and sensory sensitivity all the way to the hemiplegic aura. This finding reverberates with the fundamental discussion of whether all heterogeneous migraine symptoms share a common biological background (22–24) or if the PRS and the underlying GWAS may also capture signals of distinct genetic etiology – a question that warrants further studies. The results may also give some hint for future research on which factors in migraine could be more strongly influenced by genetics and which may potentially be driven by environmental factors.

## Limitations

Several limitations have to be considered in our study. Given that we are using a cohort of migraine families, the migraine prevalence is exceptionally high. It is also probable that the polygenic burden in non-affected family members differs systematically from the non-migraineurs in the general population. However, we could expect the shared polygenic background among family members to be more similar than observed in the general population, and thus bias our estimates of differences only downwards. Nevertheless, given this is a large Finnish family study, the generalisability of our results have to be considered carefully in a proper context. We cannot exclude the possibility that migraine manifesting in families represent some form of clustering migraine. To provide some perspective, we present our results on a scale projected to the distribution of the general Finnish population. It should be also underlined that results reflect mean differences between large groups of individuals and should not be interpreted at the individual level (19,25).

The original GWAS results (i.e. SNP weights) that are used as a foundation for the PRS are based on clinical or self-reported migraine in agreement with the ICHD-3 criteria, and presented results could be, at the first glance, considered expected and circular to some extent. However, the PRS was constructed with an independent subset of the original GWAS that explicitly excluded all studies of Finnish ancestry. Thus, it is reasonable to assume that any signals that get carried via SNP weights to our study have biological underpinning. Second, our results yield interesting observations beyond the expected correlation between symptoms and diagnosis: The relative association of symptoms can be observed and ranked as their strength of correlation with the PRS varies. Third, we saw a concordant increasing trend in PRS all the way from “No headache” up to “Hemiplegic migraine” which we would not expect if the score was strongly and circularly overfitting to the ICHD-3 MwoA, the dominating case definition in the original GWAS. Moreover, the trend of increasing number of reported headache symptoms along with the increasing PRS could be seen even among participants with no migraine headache diagnosis. As some of the GWAS studies are from self-reported migraine, it is possible that the PRS includes minor signals arising from non-migrainous headache.

Lastly, still another caveat of our study might be the use of questionnaire data instead of clinical interviews. Prior studies in FMGP suggest that our extensive questionnaire is, in general, non-inferior to office visits in a highly motivated family setting. Furthermore, it would be rather challenging to collect a study of this size based on comprehensive face-to-face neurologist interviews.

## Conclusion

In light of our results, the ICHD-3 criteria derived by the IHS align remarkably well with the polygenic risk score, a genomic biomarker of common variant burden of migraine. In our large family study, all the ICHD-3 criteria (from A to D), and all the individual symptoms they involve (number of attacks, duration, headache characteristics, nausea, vomiting, light and sound sensitivity) correlate consistently with the polygenic risk. On the PRS scale, the complex migraine phenotype shows a clear continuum from individuals with no headache to non-migrainous headache and up to patients with attacks manifesting all the features defined by the ICHD-3 headache and aura. Detailed phenotyping in a restricted, controllable, and consistent family setting can add interesting particulars to comprehensive studies that typically involve tens of thousands of participants with a generic diagnosis in the general population.

Ongoing larger GWAS studies and continuously evolving methods to derive increasingly accurate polygenic risk scores open new interesting possibilities to investigate more detailed relationship of polygenic burden to migraine characteristics and comorbidities in the future.

## Article highlights

- The ICHD-3 diagnostic criteria correlated consistently with the polygenic risk score (PRS), a genomic biomarker of common variant burden of migraine.
- The more typical and complex migraine, the higher PRS we saw on average.
- All individual diagnostic symptoms underlying the ICHD-3 criteria associated with increasing PRS and could be ranked by the strength of their association.
- Correlation of sub-diagnostic headache and PRS implies that the genetic link, if any, between tension-type headache and migraine should be studied further.

## Supplemental Material

sj-pdf-1-cep-10.1177_03331024211045651 - Supplemental material for Polygenic risk provides biological validity for the ICHD-3 criteria among Finnish migraine families Click here for additional data file. Supplemental material, sj-pdf-1-cep-10.1177_03331024211045651 for Polygenic risk provides biological validity for the ICHD-3 criteria among Finnish migraine families by Paavo Häppölä, Padhraig Gormley, Marjo E Nuottamo, Ville Artto, Marja-Liisa Sumelahti, Markku Nissilä, Petra Keski-Säntti, Matti Ilmavirta, Mari A Kaunisto, Eija I Hämäläinen, Samuli Ripatti, Matti Pirinen, Maija Wessman, Aarno Palotie, Mikko Kallela and International Headache Genetics Consortium (IHGC) in Cephalalgia
