# Supplementary material for: Polygenic risk provides biological validity for the ICHD-3 criteria among Finnish migraine families
Source: Cephalalgia. 2021 Oct 14;42(4-5):345–56. doi: 10.1177/03331024211045651 (PMC8988286; doi:10.1177/03331024211045651)
Supplement: sj-pdf-1-cep-10.1177_03331024211045651 - Supplemental material for Polygenic risk provides biological validity for the ICHD-3 criteria among Finnish migraine families [file sj-pdf-1-cep-10.1177_03331024211045651.pdf]

ICHD-3 headache criteria	Description
**Criteria A**	At least five attacks (fulfilling other criteria)
**Criteria B**	Headache attacks lasting 4-72 hr (untreated or unsuccessfully treated)
**Criteria C**	Headache has at least two of the following four characteristics:<br>1. unilateral location<br>2. pulsating quality<br>3. moderate or severe pain intensity<br>4. aggravation by or causing avoidance of routine physical activity (e.g. walking or climbing stairs)
**Criteria D**	During headache at least one of the following:<br>1. nausea and/or vomiting<br>2. photophobia and phonophobia
**- Criteria D1**	Nausea or vomiting
**- Criteria D2**	Photophobia and phonophobia

Supplemental Table 1. The IHS (International Headache Society), ICHD-3 (International Classification of Headache Disorders, 3rd edition) criteria of migraine [2].
